# *Candida duobushaemulonii*: An Old But Unreported Pathogen

**DOI:** 10.3390/jof6040374

**Published:** 2020-12-17

**Authors:** Irene Jurado-Martín, Cristina Marcos-Arias, Esther Tamayo, Andrea Guridi, Piet W. J. de Groot, Guillermo Quindós, Elena Eraso

**Affiliations:** 1 Department of Immunology, Microbiology and Parasitology, Faculty of Medicine and Nursery, University of the Basque Country, UPV/EHU, P.O. Box 699, 48080 Bilbao, Spain; irenejurado06@gmail.com (I.J.-M.); cristina.marcos@ehu.eus (C.M.-A.); esther.tamayo@ehu.eus (E.T.); andrea.guridi@ehu.eus (A.G.); guillermo.quindos@ehu.eus (G.Q.); 2 Regional Center for Biomedical Research, Laboratory for Molecular Mycology, Castilla-La Mancha Science & Technology Park, University of Castilla-La Mancha, Calle Almansa 14, 02008 Albacete, Spain; piet.degroot@uclm.es

**Keywords:** *Candida haemulonii* complex, *Candida duobushaemulonii*, molecular identification, misidentification

## Abstract

Candidiasis caused by species of the *Candida haemulonii* complex (*Candida haemulonii* and *Candida duobushaemulonii*) and closely related species, *Candida auris* and *Candida pseudohaemulonii* are increasing. These species often show reduced susceptibility to antifungal drugs, such as azoles and amphotericin B or, less frequently, echinocandins. However, conventional phenotypic identification methods are unable to accurately differentiate these species and, therefore, their prevalence may have been underestimated. In this study, 150 isolates that were probably misidentified were reanalyzed using two novel PCR approaches. We found that one isolate previously identified in 1996 as *Candida intermedia* was *C. duobushaemulonii*, being one of the oldest isolates of this species described to date. We also found that this isolate had reduced susceptibility to fluconazole, itraconazole, and amphotericin B.

## 1. Introduction

The genus *Candida* is demonstrated to be genetically heterogeneous. For that reason, many *Candida* species belong to a complex formed by various cryptic species that are phenotypically identical, and only molecular-based methods guarantee their accurate identification [1]. This is the case for *Candida haemulonii*, an opportunistic pathogen whose first clinical isolation, from a blood sample, was described in 1984 [2]. Prior to 2012, *C. haemulonii* was separated into two genetically distinct groups based on isoenzyme and protein profiles and DNA relatedness studies [3]. However, in the same year, as further studies revealed the heterogeneity in the DNA sequence of various genes, the complex was reclassified as two species (*C. haemulonii sensu stricto* and *Candida duobushaemulonii*) and one variety (*C. haemulonii* var. *vulnera*) [4]. Concurrently, two new species phylogenetically close to *C. haemulonii* complex, namely, *Candida pseudohaemulonii* and *Candida auris*, were described in 2006 and 2009, respectively [5,6].

Although infections caused by this complex of species remain rare, since they are not usually involved in outbreaks but in sporadic infections [7,8,9], they are increasingly being isolated worldwide and are reported as important fungi in clinical settings [10,11,12,13]. However, the case of *C. auris* stands out. Since its first description in 2009 in Japan, this fungus has been identified worldwide in a short time span, suggesting rapid dissemination. As of today, Spain, the United Kingdom, India, Kuwait and USA are among the world countries with the highest number of reported cases (>100) [14,15]. Infections by *C. auris* cause a wide range of clinical manifestations, but it has mostly been isolated from blood and other deep-seated locations. Moreover, this yeast is associated with nosocomial outbreaks and an estimated high in-hospital mortality rate (30–72%) [14,15].

Both species of the *C. haemulonii* complex and its relatives *C. auris* and *C. pseudohaemulonii* frequently show in vitro resistance to amphotericin B and fluconazole, which often leads to clinical failure [4,5,10,16,17,18,19]. Although less common, low susceptibility to echinocandins has also been reported [4,18,20,21,22]. Besides, accurate identification of these species is troublesome, as conventional panels used in routine microbiology laboratories frequently misidentify them. These species are mistaken not only for other *Candida* species, but also with close genera, such as *Saccharomyces* and *Rhodotorula* [23]. As a result, these pathogens are often unreported as causal agents of fungal infections and, consequently, their actual incidence and global prevalence might be underestimated [24,25].

Currently, only matrix-assisted laser desorption/ionization time-of-flight mass spectrometry (MALDI-TOF MS) and molecular techniques, such as sequencing of genetic loci of the ribosomal DNA¸ including the D1/D2 domain, internal transcribed spacer (ITS) regions, and *RPB1* and *RPB2*, have been demonstrated to provide reliable identification of these species [16,18]. However, these techniques are not available in most routine microbiology laboratories, and there may be issues when testing mixed clinical samples, as they could be not able to distinguish between the species involved [26]. Aiming to achieve simple and easily accessible techniques for accurate identification of *C. auris*, various species-specific conventional or real-time PCR-based assays have been developed. These assays involve amplification of different regions of the ribosomal gene complex [27,28,29,30] or, alternatively, unique glycosylphosphatidylinositol (GPI) protein-encoding genes [31]. Likewise, given the growing importance of the complex, other groups have come up with assays that allow discrimination between *C. auris* and its closest relatives [27,32,33,34].

In light of the above, the aim of this study was to deepen our knowledge about the prevalence of this emergent, multidrug-resistant complex of cryptic *Candida* species by surveying our culture collection for previous potentially misidentified clinical isolates using novel molecular techniques.

## 2. Materials and Methods

### 2.1. Fungal Isolates

This study includes 150 clinical isolates from the yeast stock collection of the Medical Mycology Laboratory at the University of the Basque Country (UPV/EHU), collected between 1993 and 2014, which are likely to have been inaccurately identified. These isolates were previously identified by API^®^ID 32C (bioMérieux, Marcy l’Etoile, France) biochemical panel as: *Candida famata* (8), *C. famata*/*Candida guilliermondii* (8), *C. guilliermondii*/*C. famata* (8), *C. haemulonii* (8), *Candida intermedia* (8), *Candida lipolytica* (8), *Candida lusitaniae* (8), *C. lusitaniae*/*Candida pulcherrima* (8), *Candida rugosa* (8), *Candida tropicalis* (8), *Candida kefyr* (7), *Candida valida* (7), *Candida pelliculosa* (6), *Candida lambica* (4), *Candida nivariensis* (4), *Candida sake* (4), *Candida* spp. (4), *Candida zeylanoides* (4), *Candida colliculosa* (3), *Candida curvata* (2), *Candida holmii* (2), *Candida magnoliae* (2), *Candida norvegensis*/*Candida inconspicua* (2), *Candida rubra* (2), *Candida stellatoidea* (2), *Debaryomyces polymorphus* (2), *Saccharomyces cerevisiae* (2), *Candida catenulata* (1), *Candida glabrata* (1), *C. guilliermondii* (1), *C. lusitaniae*/*C. tropicalis* (1), *Candida norvegensis* (1), *C. pulcherrima*/*C. lusitaniae* (1), *C. pulcherrima* (1), *Candida quercitrusa* (1), *Candida silvicola* (1), *Candida sake*/*C. tropicalis* (1), and *Candida utilis* (1). Additionally, as positive controls, we included two *C. auris* and one *C. duobushaemulonii* clinical isolates from Hospital La Fe (Valencia), already confirmed by ITS sequencing, and a *C. pseudohaemulonii* reference strain (CBS 10004).

### 2.2. Sample Preparation and DNA Extraction

Both clinical isolates and the reference strain were cultured on Sabouraud dextrose agar (SDA) or yeast extract peptone dextrose (YEPD) medium at 37 °C during 24–48 h. Then, DNA extraction was performed using the DNeasy^®^ UltraClean^®^ Microbial Kit (QIAGEN, Hilden, Germany) following the manufacturer’s instructions. DNA purity and quantity were assessed using a NanoDrop ND-1000 spectrophotometer (Thermo Fisher Scientific, Waltham, MA, USA).

### 2.3. PCR-Based Molecular Identification

**Figure 1:**
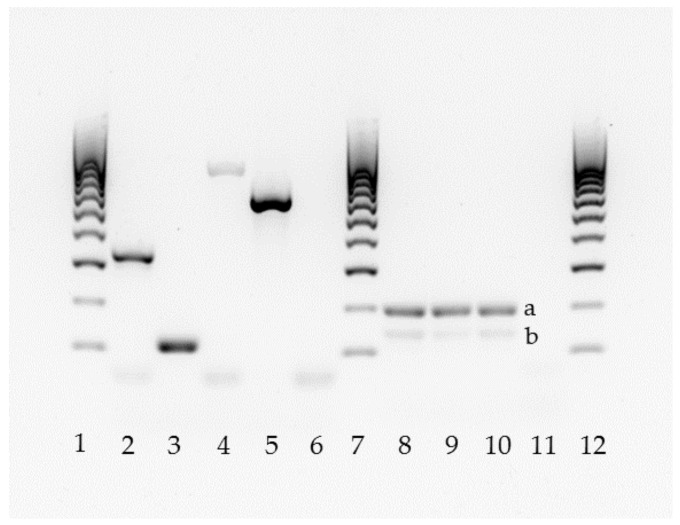
Molecular identification of the *C. haemulonii* complex by two PCR methods (lanes 2–6 [32]; lanes 8–11 [35]). Lanes 1, 7, and 12: 100 bp DNA ladder. Lane 2: *C. auris*; 3: *C. duobushaemulonii*; 4: *C. haemulonii*; 5: *C. pseudohaemulonii*; 6: negative control. Lanes 8–10: *C. auris* (**a**), 5.8S rDNA band (**b**); 11: negative control without DNA.

The 150 isolates and the four positive controls were analyzed by two different PCR techniques, using primers and amplification conditions described by Ruiz-Gaitan et al. [35] and Arastehfar et al. [32] (Table 1). One was a duplex *C. auris*-specific PCR that also amplifies the 5.8S rDNA gene as quality control band [35] and the other was a *C. haemulonii* complex-tetraplex PCR [32] (Figure 1).

### 2.4. ITS Region Sequencing and Species Identification

When robust amplification in any of the PCR or low-intensity bands were detected, sequencing of the ITS1-5.8S-ITS2 gene was performed for confirmation. This region was amplified with ITS1 and ITS4 primers as described previously [36]. Unpurified PCR products were sent to the Advanced Research Facilities of the University of the Basque Country (SGIker), where samples were purified and sequenced. Received sequences from both DNA strands were analyzed by Chromas v. 2.6.4 (Technelysium Pty Ltd., South Brisbane, Australia) and BioEdit v. 7.2.6.1 (Tom Hall, Raleigh, NC, USA), and sequence identity for species identification was determined using the Basic Local Alignment Search Tool (BLAST) at the NCBI database (https://blast.ncbi.nlm.nih.gov/Blast.cgi).

### 2.5. In Vitro Antifungal Susceptibility

In vitro susceptibility testing of the identified isolates related to the *C. haemulonii* complex (*n* = 9) as well as the positive controls (*n* = 4) against nine different antifungal agents (anidulafungin, micafungin, caspofungin, posaconazole, voriconazole, itraconazole, fluconazole, amphotericin B, and 5-fluorocytosine) was tested using SensititreTM YeastOne YO10 (Thermo Scientific, Waltham, MA, USA), following the manufacturer’s instructions. *Candida parapsilosis* ATCC 22019 and *C. krusei* ATCC 6258 were included as quality controls.

### 2.6. Phenotypical Characterization

All the isolates belonging to the *C. haemulonii* complex (*n* = 13) were plated on CHROMagar *Candida* medium supplemented with Pal’s medium at both 37 °C and 42 °C during 24–48 h, in order to assess growth ability and colony characteristics [37].

## 3. Results

### 3.1. Molecular Identification

Except for the two confirmed *C. auris* clinical isolates, no other *C. auris* was found using the *C. auris*-specific PCR. Nevertheless, as the quality control band of 5/8 *C. rugosa*, 7/8 *C. lipolytica*, 1/2 *C. magnoliae*, and 1/8 *C. intermedia* was absent, ITS sequencing was performed for these isolates. The identification of the seven *C. lipolytica* and the only *C. magnoliae* isolate was confirmed. On the contrary, the five *C. rugosa* and the only *C. intermedia* were all re-identified as *Candida pararugosa* (Table 2). The *C. haemulonii* complex-tetraplex PCR method accurately identified the four isolates used as positive controls. Neither *C. auris* nor *C. pseudohaemulonii* were detected in the collection and the eight isolates previously identified as *C. haemulonii* were confirmed by the tetraplex PCR, as they all produced a 696 bp sized band. Interestingly, one isolate identified in 1996 as *C. intermedia* by API^®^ID 32C produced a 115 bp sized band, corresponding to *C. duobushaemulonii*. Additionally, we re-analyzed this isolate by the current API^®^ID 32C panel, which identified it as *C. sake* (87.4%, acceptable profile). The eight *C. haemulonii* isolates, the new *C. duobushaemulonii*, as well as the four positive controls, were all confirmed by ITS sequencing showing a percentage of identity with the reference strains higher than 99%.

### 3.2. Fungal Susceptibility

Although the *C. duobushaemulonii* isolate that was used as positive control showed susceptibility to all azole agents, the re-identified *C. duobushaemulonii* isolate was less susceptible to itraconazole (MIC = 0.5 μg/mL) and fluconazole (MIC = 32 μg/mL). Six *C. haemulonii* isolates also showed a lower susceptibility to itraconazole (MIC = 0.5 μg/mL) and fluconazole (MIC = 16–32 μg/mL), other one was inhibited by 1 μg/mL of itraconazole, and another one by 64 μg/mL of fluconazole, and another showed reduced susceptibility to both agents (MIC = 1 and 64 μg/mL, respectively, of itraconazole and fluconazole). Both *C. auris* isolates were less susceptible to fluconazole (MIC > 256 μg/mL) and itraconazole (MIC = 0.25 μg/mL). One of them also showed decreased susceptibility to voriconazole (MIC = 2 μg/mL). With regard to amphotericin B, all but one *C. haemulonii* isolate showed reduced susceptibility (MIC > 2 μg/mL). Finally, no reduced susceptibility was found to either echinocandins or 5-flucytosine (Table 2).

### 3.3. Phenotypic Characteristics

**Figure 2:**
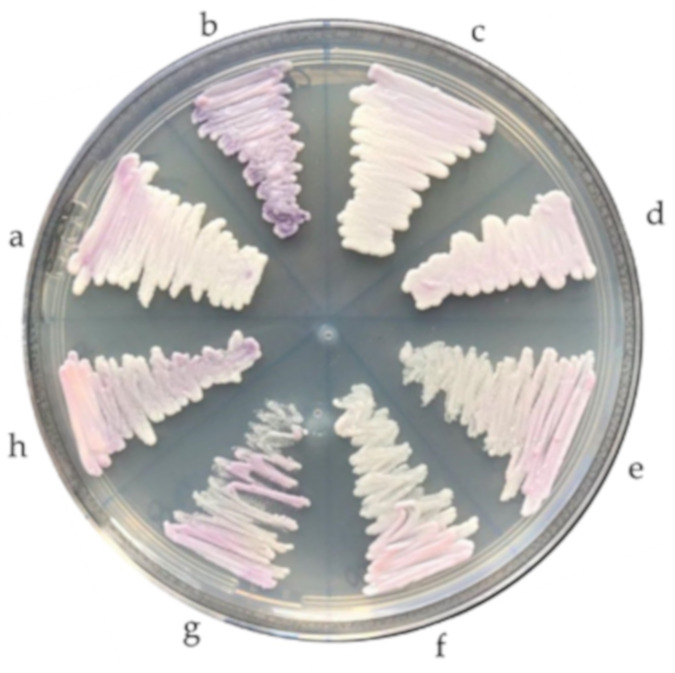
Phenotypic characteristics of *Candida haemulonii* complex species grown on CHROMagar Candida medium: (**a**) *Candida duobushaemulonii*, (**b**) *Candida pseudohaemulonii*, (**c**,**d**) *Candida auris,* and (**e**–**h**) *Candida haemulonii*.

When grown on chromogenic agar, all but the *C. pseudohaemulonii* reference strain, whose colonies were purple, showed smooth, matte, and light-pink colored colonies (Figure 2). When cultured at 42 °C, only the two *C. auris* isolates were able to grow.

## 4. Discussion

Although *Candida albicans* remains the most often isolated species, a remarkable shift in the etiology of candidiasis is ongoing, as previously uncommon non-*C. albicans* species are arising or increasing in prevalence [38]. Part of this increase in prevalence of uncommon species of *Candida* might be due to the broadened use of molecular methods, such as PCR and MALDI-TOF for identification of clinical isolates. Examples of such pathogens are species related to the *C. haemulonii* complex, whose frequency has increased over the years. Between 1997 and 2007, the prevalence *of C. haemulonii* was very low (<0.01%), as observed by the ARTEMIS DISK Global Surveillance Study [39]. In a different retrospective study that was carried out between 2001 and 2005, this species was the sixth most common *Candida* species isolated (1.5%). Ramos et al. reported that *C. haemulonii* was the fourth most common species (12.1%) causing cutaneous candidiasis in Brazil from 2008 to 2009 [10]. A study in India reported that the prevalence of this species increased from 5.5% in 2006 to 18.2% in 2008 [40]. However, the identifications in those studies were performed using commercially available biochemical methods, such as API, MicroScan, and Vitek, which have been proved to misidentify these species. In addition, *C. duobushaemulonii* was not described until 2012, when Cendejas-Bueno et al. established *C. haemulonii* as a complex of species [4]. Therefore, whether candidiasis caused by these species is relatively new, as suggested by Frías-de-León et al. [13], or longstanding but unidentified remains unknown. Our results support this latter idea. We carried out a retrospective study in which we surveyed for species related to this complex in a culture collection that contains clinical isolates from 1993 to 2014 using novel molecular methods. From that year onwards, all the isolates with questionable identifications were sequenced. Interestingly, we discovered that one isolate that was identified in 1996 as *C. intermedia* by API^®^ID 32C in fact was *C. duobushaemulonii*, and this type of misidentification has not been described before. In accordance with literature, this result shows the inaccuracy of biochemical methods towards correct identification of some infrequent *Candida* species and, consequently, that the real clinical incidence of the *C. haemulonii* complex might have been underestimated. For instance, Ramos et al. found that four *C. haemulonii* isolates that were collected between 2009 and 2013 in fact were *C. duobushaemulonii* [10,23].

Moreover, we show that the current API^®^ID 32C panel is still unable to identify this species, which reinforces the importance of using molecular methods or MALDI-TOF to detect pathogens related to the complex. The PCR proposed by Ruiz-Gaitán et al. [31] that was tested in our study accurately identified both *C. auris* isolates and did not yield any *C. auris*-specific amplicon with other tested species. The 5.8S rDNA control amplicon was not produced with *C. pararugosa*, *C. magnoliae*, and *C. lipolytica* isolates. Accordingly, Ruiz-Gaitán et al. already reported that these control primers do not yield amplicons and therefore cannot be used in more distinct species such as *C. lipolytica* [31], which we confirmed in our study. However, as there are no studies of reported misidentifications of *C. auris* with those three species and their incidence of causing candidiasis is utterly low, we believe this PCR design is a powerful approach for clinicians to effectively detect *C. auris* infections. Likewise, the tetraplex-PCR proposed by Arastehfar et al. [32] is also an accurate and useful molecular technique. This was also demonstrated by Frias-de-León et al. [13], who showed correct identification of four *C. auris* control isolates, eight *C. haemulonii* isolates, and two *C. duobushaemulonii*, whereas no other isolate yielded any amplicon.

The re-identified *C. duobushaemulonii* isolate in the present study was obtained in 1996 from a toenail of a patient from Bizkaia (Spain), while the eight *C. haemulonii* isolates had been obtained in 2000 from various sources, including both invasive and superficial infections. Our results support the idea that species of this complex were primarily associated to superficial infections, prior to its emergence related to invasive candidiasis. Even though *C. haemulonii* was first clinically isolated in 1984 from the blood of a patient [2], most of the invasive infections caused by species belonging to this complex were reported in more recent years. In 1991, Gargeya et al. reported onychomycoses cases in the USA caused by *C. haemulonii* [41], and the five isolates used by Lehmann et al. in 1993 were also obtained from skin and nails [3]. In the study carried out by Ramos et al., all the isolates obtained from non-invasive sources (nails and skin) were collected prior to 2009, whereas all isolates collected between 2010 and 2013 originated from invasive sites [10]. Likewise, Hou et al. collected 31 clinical isolates (26 *C. haemulonii* and five *C. duobushaemulonii*) from 2010 to 2014, which were all recovered from invasive sources, mainly from blood and cerebrospinal fluid [22]. Although Lehmann et al. had already described in 1993 that *C. haemulonii* was a complex of two genetically different groups (I and II) [3], our re-identified isolate, together with the first isolate from a foot ulcer in 1990 [42] are, to our knowledge, the oldest *C. duobushaemulonii* isolates found. This underlines the importance of our study to understand the origin and spread of cryptic emergent species.

The *C. duobushaemulonii* isolate re-identified in our study, the eight *C. haemulonii* isolates and the two control *C. auris* isolates had reduced susceptibility to fluconazole and itraconazole, whereas seven *C. haemulonii* isolates had also reduced susceptibility to amphotericin B. This concurs with other studies, as clinical isolates of this complex usually show in vitro reduced susceptibility or resistance to these antifungal drugs [4,5,10,16,17,18,19], often being associated with clinical failure [4,10,17]. Recently, a potential mechanism of resistance among the species of *C. haemulonii* complex to amphotericin B has been described. The membrane of the species of the complex is composed mainly of ergosterol pathway intermediates, thus there is no target for amphotericin B [43]. As all clinical isolates were susceptible to echinocandins, these antifungal drugs might be the best treatment option for infections caused by species related to the complex, as was also suggested elsewhere [12,17]. Nevertheless, the tendency of the complex to show resistance to azoles, echinocandins, and amphotericin B reinforces the necessity to accurately identify these uncommon species.

Finally, morphological characteristics such as colony color are not very useful for the identification of these species. Although in our study *C. haemulonii*, *C. auris*, and *C. duobushaemulonii* isolates showed smooth, light-pink colored colonies and the *C. pseudohaemulonii* isolate purple colonies on CHROMagar, other authors have reported that *C. haemulonii* complex species and *C. auris* can also show a dark pink color [21]. On the other hand, the ability of only *C. auris* isolates to grow well at 42 °C, which has also been described before [10,18,37], could comprise a helpful characteristic to differentiate *C. auris* from the other species.

## 5. Conclusions

Species of the *C. haemulonii* complex are emerging invasive pathogens that often show resistance to commonly used antifungal drugs. Therefore, their accurate and quick identification using novel molecular methods is essential as they can cause challenging invasive infections that pose difficulties in their therapeutic management. Although initially described as etiological agents of superficial candidiasis, surveillance should be conducted to clarify the mechanisms involved in developing invasive candidiasis.

## Figures and Tables

**Table 1:** Primers used in this study for each PCR-based method and expected amplicon size.

Primer Name	Primer Sequence (5′ 3′)	Amplicon Size (bp)	Reference
05701_F	GCAGCACTCGTGAGAGAACT	193	[35]
05701_R	GGCTGGTTCTCCTGCTCATT		
RDN58_F	GGATCTCTTGGTTCTCGC	134	
RDN58_R	CGCTCAAACAGGCATGC		
Uni-F	GAACGCACATTGCGCCTTGG		[32]
Au-R	TCCAAAGGACTTGCCTGCT	331	
Du-R	GTAGACTTCGCTGCGGATATGTTA	115	
Ha-R	ATTGCGCCAGCATCCTTATTG	696	
Ps-R	GCACCCGATGCTGACAGTCTAC	576	

**Table 2:** Profiles of identification and susceptibility of clinical and control isolates of *C. haemulonii* complex against nine antifungal agents.

Isolate Number	Year of Isolation	Source of Isolation	Previous Identification (API ID^®^32C)	*C. auris*-Specific PCR		*C. haemulonii* Complex-Tetraplex PCR	ITS Sequencing	MIC (μg/mL)/Susceptibility Profile								
				*C. auris*- Specific Band	Quality Control Band			AND	MCF	CAS	POS	VOR	ITR	FLZ	AB	5FL
00-093	2000	Maxilary sinus	*C. haemulonii*	-	+	*C. haemulonii*	*C. haemulonii*	0.125	0.06	0.25	0.25	0.25	0.5 ^RS^	16 ^RS^	4 ^RS^	<0.06
00-094	2000	Homograft liquid	*C. haemulonii*	-	+	*C. haemulonii*	*C. haemulonii*	0.03	0.06	0.125	0.25	0.25	0.5 ^RS^	16 ^RS^	2 ^RS^	<0.06
00-095	2000	Adenophlegmon liquid	*C. haemulonii*	-	+	*C. haemulonii*	*C. haemulonii*	0.125	0.125	0.25	0.5	0.25	0.5 ^RS^	16 ^RS^	4 ^RS^	<0.06
00-096	2000	Bronchoalveolar lavage	*C. haemulonii*	-	+	*C. haemulonii*	*C. haemulonii*	0.125	0.125	0.25	0.25	0.5	0.5 ^RS^	16 ^RS^	4 ^RS^	<0.06
00-097	2000	Corneal abscess	*C. haemulonii*	-	+	*C. haemulonii*	*C. haemulonii*	0.125	0.125	0.25	0.25	0.5	0.5 ^RS^	64 ^RS^	>8 ^RS^	<0.06
00-180	2000	Catheter	*C. haemulonii*	-	+	*C. haemulonii*	*C. haemulonii*	0.125	0.125	0.25	0.5	1	1 ^RS^	64 ^RS^	>8 ^RS^	0.125
00-181	2000	Peritoneal fluid	*C. haemulonii*	-	+	*C. haemulonii*	*C. haemulonii*	0.125	0.125	0.25	0.25	0.5	0.5 ^RS^	16 ^RS^	1	<0.06
00-182	2000	Mouth	*C. haemulonii*	-	+	*C. haemulonii*	*C. haemulonii*	0.06	0.125	0.25	0.5	0.5	1 ^RS^	32 ^RS^	4 ^RS^	<0.06
96-013	1996	Toe nail	*C. intermedia*	-	+	*C. duobushaemulonii*	*C. duobushaemulonii*	0.125	0.06	0.06	0.25	0.25	0.5 ^RS^	32 ^RS^	1	<0.06
19-038				*-*	+	*C. duobushaemulonii*	*C. duobushaemulonii*	0.25	0.06	0.03	0.03	0.03	0.125	2	1	<0.06
17-213	2017	Blood		*C. auris*	+	*C. auris*	*C. auris*	0.125	0.06	0.125	0.06	1	0.25 ^RS^	>256 ^RS^	0.5	<0.06
17-257	2017	Blood		*C. auris*	+	*C. auris*	*C. auris*	0.125	0.06	0.125	0.125	2 ^RS^	0.25 ^RS^	>256 ^RS^	1	<0.06
CBS 10004				*-*	+	*C. pseudohaemulonii*	*C. pseudohaemulonii*	0.125	0.06	0.06	0.015	0.03	0.03	2	0.125	<0.06

AND, anidulafungin; MCF, micafungin; CAS, caspofungin; POS, posaconazole; VOR, voriconazole; ITR, itraconazole; FLZ, fluconazole; AB, amphotericin B; 5FL, 5-flucytosine. RS, reduced susceptibility.
